# The role of cardiac troponin I as a prognosticator in critically ill medical patients: a prospective observational cohort study

**DOI:** 10.1186/cc3731

**Published:** 2005-05-31

**Authors:** Daniel A King, Shlomi Codish, Victor Novack, Leonid Barski, Yaniv Almog

**Affiliations:** 1 Medical Intensive Care Unit, Soroka University Medical Center and the Faculty of Health Sciences, Ben-Gurion University, Beer Sheva, Israel

## Abstract

**Introduction:**

Myocardial injury is frequently unrecognized in intensive care unit (ICU) patients. Cardiac troponin I (cTnI), a surrogate of myocardial injury, has been shown to correlate with outcome in selected groups of patients. We wanted to determine if cTnI level measured upon admission is an independent predictor of mortality in a heterogeneous group of critically ill medical patients.

**Methods:**

We conducted a prospective observational cohort study; 128 consecutive patients admitted to a medical ICU at a tertiary university hospital were enrolled. cTnI levels were measured within 6 h of admission and were considered positive (>0.7 ng/ml) or negative. A variety of clinical and laboratory variables were recorded.

**Results:**

Both cTnI positive and negative groups were similar in terms of age, sex and pre-admission co-morbidity. In a univariate analysis, positive cTnI was associated with increased mortality (OR 7.0, 95% CI 2.44–20.5, p < 0.001), higher Acute Physiology and Chronic Health Evaluation (APACHE) II scores and a higher rate of multi-organ failure and sepsis. This association between cTnI and mortality was more pronounced among elderly patients (>65 years of age). Multivariate analysis controlling for APACHE II score revealed that elevated cTnI levels are not independently associated with 28-day mortality.

**Conclusion:**

In critically ill medical patients, elevated cTnI level measured upon admission is associated with increased mortality rate. cTnI does not independently contribute to the prediction of 28-day mortality beyond that provided by APACHE II.

## Introduction

Assessing the severity of illness and outcome of critically ill patients is important as it influences management strategies and resource allocation. Historically, research aimed at determining factors associated with intensive care unit (ICU) mortality focused on individual risk factors and the development of multivariable prediction scores. These investigations consistently suggested the importance of organ system failure as strong predictors of both ICU and hospital mortality [1-4]. Over the past decade, several studies indicated that cardiac dysfunction is a frequent and important factor in determining the outcome of critically ill patients [5,6]. The pathophysiology of myocardial injury in critically ill patients is believed to be multifactorial, including the underlying disease process, hypoxemia and acidosis as well as therapeutic maneuvers [7,8]. It is estimated that as many as 15% of ICU admissions are complicated by some degree of myocardial injury and as many as 85% of patients with sepsis may have raised cardiac troponin [5,9]. Elevated serum levels of cardiac troponin I (cTnI), a myocardial regulatory protein of the thin actin filament, are considered highly sensitive and specific indicators of myocardial injury [10]. Serial measurement of cTnI is routinely used in the evaluation of patients with acute coronary syndromes (ACS) for diagnostic and prognostic purposes [10-12]. Several studies have assessed the prognostic value of elevated cTnI in critically ill patients without ACS. While some suggested that cTnI levels correlate with myocardial damage and poor outcome, others could not confirm this association [6,13-17]. Because cTnI elevation reflects organ failure (i.e. myocardial injury) its role as an additional marker of severity of illness and outcome is biologically plausible; however, limited information is available regarding the relative significance of cTnI elevation as an independent predictor of mortality in relation to the Acute Physiology And Chronic Health Evaluation (APACHE) II score. We hypothesized that elevated cTnI will not contribute to the mortality prediction provided by the multivariable APACHE II score. Therefore, we conducted a prospective cohort study in which the main purpose was to determine whether cTnI, measured upon admission, is an independent predictor of mortality in a heterogeneous group of critically ill medical patients.

## Materials and Methods

### Study location and population

The study was conducted within the medical ICU (MICU, eight beds) of Soroka University Medical Center, Beer-Sheva, Israel, a tertiary university hospital in Israel. All patients admitted to the MICU during a nine-month period (September 2002 to June 2003) were evaluated prospectively. The nursing staff and the physicians providing care for the patients in the MICU were completely blinded to the troponin results. A total of 128 consecutive patients were enrolled in this observational cohort study. All definitions were determined prospectively. The definitions used for sepsis, severe sepsis and organ failure were those used by the Recombinant Human Activated Protein C Worldwide Evaluation in Severe Sepsis (PROWESS) investigators [18]. Patients diagnosed with ACS, defined as unstable angina, typical chest pain, ischemic ECG changes or cardiogenic pulmonary edema were excluded as well as patients requiring chronic hemodialysis or patients who underwent major surgery during the month preceding admission (in order to exclude peri-operative myocardial injury). The Ethics Committee of the Soroka University Medical Center approved the study protocol prior to its initiation.

### Data collection

One of the investigators who did not participate in routine patient care made daily rounds in the ICU recording relevant data from patient medical records and the hospital mainframe computer for reports of laboratory and microbiologic data. A complete history and physical examination was recorded on each patient enrolled in the study. APACHE II score was calculated based on the worst values for the first 24 h after ICU admission. Serum levels of cTnI were measured within 6 h of admission. The commercial assay AxSYM Troponin-I (Abbott laboratories, Abbott Park, Illinois, USA), a micro particle enzyme immunoassay, was used to determine cTnI levels. The assay characteristics were as follows: detection limit 0.3 ng/ml; analytical range 0–50 ng/ml; assay coefficient of variation (CV) range 5.2–7.8%. The cTnI cutoff is 0.7 ng/ml with a CV of 10%. Thus any cTnI blood level > 0.7 ng/ml was considered abnormal and indicative of myocardial injury. The primary end point was 28-day mortality. Secondary end points included days on mechanical ventilation, length of stay, and the number of failing organs.

### Statistical analysis

Continuous variables' data are expressed as mean value ± SD. Bi-variate hypotheses involving continuous variables were tested with a t-test for independent variables with normal distribution and Mann-Whitney test for variables with abnormal distribution. Normality of the data was tested with a one-sample Kolmogorov-Smirnov test to indicate the appropriateness of parametric testing. Categorical variables are expressed as percentage; comparisons between groups were made using the chi square test. Logistic regression analysis was used to identify independent variables associated with death. Variables that were associated with mortality in univariate analysis were considered for inclusion in the model, whereas parameters already incorporated into the APACHE-II score, such as age, creatinine level and mean arterial pressure were not included. Cumulative survival curves were constructed using the Kaplan-Meier method and compared with the log-rank test. Results were considered significant at p < 0.05. Statistical analysis was performed with the SPSS 11.5 statistical package (SPSS Inc. Chicago, IL, USA).

## Results

A total of 128 patients met inclusion criteria and were evaluated during the study period. Demographic and clinical characteristics of the study patients enrolled are shown in Table 1. The mean age of the study population was 53.9 ± 19 years and 68% were male. There was no significant difference between the cTnI positive and negative groups in terms of background illnesses; ischemic heart disease (20% versus 12%, p = 0.2), chronic obstructive pulmonary disease (COPD; 17% versus 12%, p = 0.43), diabetes mellitus (31% versus 17%, p = 0.08), arterial hypertension (34% versus 26%, p = 0.34) or malignancy (6% versus 3%, p = 0.61). Stratifying the patients according to the absence or presence of cTnI elevation revealed that of the various causes of admission, only sepsis was associated with elevated troponin (p = 0.008). Patients with elevated cTnI had a significantly higher APACHE II score (p < 0.001), required longer duration of mechanical ventilation (p = 0.004) and their mortality rate increased from 9.7% to 42.9% (OR 7.0, 95%CI 2.68–18.3, p < 0.001). Clinical variables upon admission, particularly vasopressor requirement, did not correlate with cTnI levels. Although creatinine levels were higher in the cTnI group, none of the patients required renal replacement therapy.

Table 2 shows demographic and clinical information of the study population stratified according to outcome. Patients who died had higher cTnI levels (p < 0.001), were significantly older (p = 0.001), had greater APACHE II scores (p = 0.001) and longer duration of mechanical ventilation (p < 0.001). In contrast, the cause of admission was not associated with differences in mortality rate. Of the clinical variables evaluated upon admission only mean arterial pressure (p = 0.006), creatinine level (p = 0.004) and vasopressor requirement were significantly associated with higher mortality (p = 0.02).

**Figure 1:**
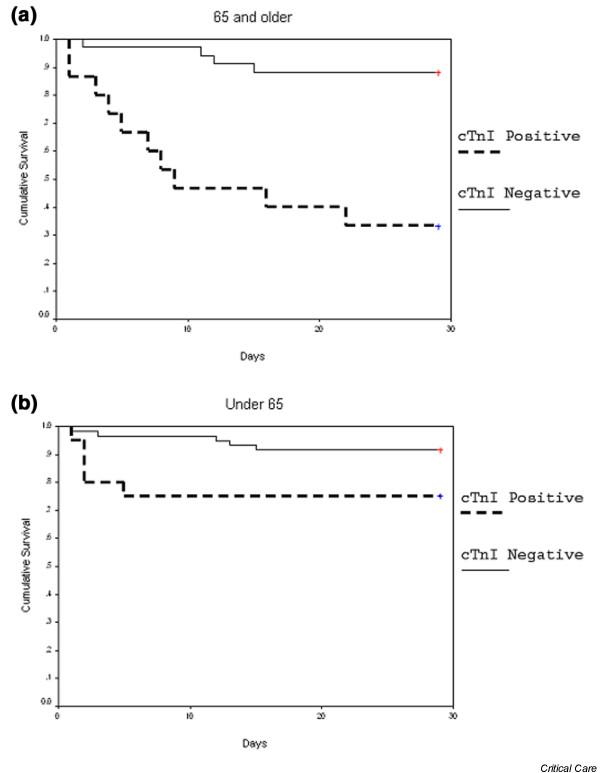
Kaplan-Meier curves showing mortality rates among patients. **(a) **65 years of age and older or **(b) **younger than 65.

Among elderly (older than 65 years) patients (n = 49) with elevated cTnI levels, the 28-day mortality rate was 10/15 patients (66.7%), while the mortality rate among elderly patients with normal cTnI levels was only 4/34 patients (11.8%). This pattern was observed also in the younger patients, but to a lesser extent. Kaplan-Meier survival analysis in the younger and older age groups is shown in Fig. 1a,b. Log-rank tests for both groups were statistically significant: 0.04 and <0.001, respectively.

The results of the multivariate analysis (logistic-regression) are shown in Table 3. The two variables that were included in the final model were cTnI and APACHE II; both highly correlated with mortality in univariate analysis. Elevated cTnI levels were not found to be an independent predictor of mortality regardless of APACHE II score (OR 2.8, 95% CI 0.87–9.2, p = 0.085). Multivariate analysis of the subgroup of patients admitted without sepsis (n = 82) reveals that while APACHE II remained significantly associated with 28-day mortality (OR 1.2, 95% CI 1.03–1.28 per point increment), abnormal cTnI level was not (OR 1.2, 95% CI 0.21–7.1).

## Discussion

We found that in critically ill medical patients, elevated cTnI is associated with increased mortality and longer duration of mechanical ventilation. cTnI does not, however, independently contribute to the prediction of 28-day mortality beyond that provided by APACHE II.

Cardiac troponin I and T are the most specific and sensitive laboratory markers of myocardial cell injury and may be elevated in patients presenting with many conditions other than acute coronary syndrome [9,11]. Elevated cTnI levels also correlate with decreased left ventricular function in both coronary and non-coronary patients [13,16]. Cardiac dysfunction during sepsis is fairly well documented and has been associated with poor prognosis [5]. Moreover, a small recent study evaluated the value of brain natriuretic peptide (BNP) plasma levels as a marker of systolic myocardial dysfunction during severe sepsis [19]. This study suggested that systolic dysfunction is present in 44% of patients with severe sepsis, BNP is useful in its detection and high plasma levels of BNP are associated with poor outcome [19]. It remains unclear though whether, in this context, elevated cTnI reflects reversible or irreversible myocardial damage [7,9]. Our data indicate that in patients over 65 there is a stronger correlation between elevated cTnI and mortality, which can probably be attributed to the extent, and possibly irreversibility, of myocardial damage in this age group. An interesting finding of this study was that most deaths among younger patients occurred within the first five days, whereas in the elderly group the majority of deaths (60%) occurred after this time frame. This data may suggest that younger patients who survive the initial insult do relatively well. Our study, however, was not designed nor powered to address the effect of age on outcome.

Several studies have addressed the prognostic value of elevated cTnI in non-coronary patients. In selected groups such as COPD and hemodyalisis patients, elevated cTnI correlated with poor outcome [20,21]. A study in emergency department patients has shown that there is a significant correlation between cTnI elevation and outcome. APACHE II is not provided, however, nor were the patients stratified by cause of admission. Therefore, no comparison between this study and ours could be performed [17]. Relos *et al*. [22], evaluating surgical ICU patients, suggested that moderate elevation of serum troponin I, which are below the threshold required to diagnose overt myocardial infarction, may reflect ongoing myocardial injury in the critically ill and are associated with a higher mortality rate and longer hospital and ICU length of stay. To the best of our knowledge, only one study suggested an independent predictive value of elevated cTnI after controlling for severity of illness assessed by APACHE II [23]. A strong correlation between mortality and elevated cTnI in critically ill medical patients without coronary disease was shown in this study. The sample size was rather small (58 patients), however, and the majority of patients had sepsis (88%), which limits the interpretation of these results. In contrast to these studies, Kollef *et al*. [6] suggested that serial measurements of cTnI do not independently contribute to the prediction of hospital mortality beyond that provided by clinically recognized cardiac dysfunction. Differences in design and patient mix preclude meaningful comparisons between this study and ours. Our observation that cTnI elevation is not an independent predictor of mortality is not surprising because troponin reflects a single system malfunction while the multivariable APACHE II reflects several highly relevant systems in the context of critically ill patients. It is, therefore, unlikely that a single assay will provide an independent additional value beyond that provided by APACHE II. Nonetheless, our finding that cTnI elevation is an important marker of severity of illness and is associated with high mortality rate is still clinically relevant, particularly in view of the fact that the Kaplan-Meier analysis indicates that the discriminative effect of cTnI elevation is evident from the first day.

The present study included a relatively small number of patients, limiting the significance of post-hoc subgroup analysis and our ability to identify other independent determinants of early mortality. The fact that the frequency of ischemic heart disease (IHD) was similar among cTnI positive patients and cTnI negative patients supports the assumption that the elevated cTnI in our study should not be attributed to ACS. As we did not systematically perform echocardiography or evaluation of coronary flow in these patients, more objective assessment of coronary anatomy and myocardial function is not available. Therefore, any correlation between cTnI levels, in these patients, and irreversible myocardial dysfunction or ACS remains deductive. As indicated earlier, however, elevated cTnI has been previously shown to correlate with left ventricular function. In our study, cTnI was sampled only once upon admission. Even though the time course and kinetics of cTnI and its relation to outcome may be of interest, the main purpose of our study was to determine whether early cTnI elevation is of clinically relevant importance.

## Conclusion

We conclude that troponin elevation may be used as an early marker of severity of illness and outcome, particularly in older patients, but it is not an independent predictor of mortality. Additional larger prospective studies will be required to determine if a single serum marker, reflecting myocardial injury, could be established as an independent prognostic tool.

## Key messages

• 	cTnI is a surrogate of myocardial injury in critically ill medical patients

• 	cTnI may be used as an early marker of outcome

• 	The correlation between elevated cTnI and mortality may be stronger among patients older than 65 years of age

• 	cTnI does not independently contribute to the prediction of 28-day mortality beyond that provided by APACHE II.

## Abbreviations

ACS = acute coronary syndromes; APACHE = Acute Physiology and Chronic Health Evaluation; BNP = brain natriuretic peptide; COPD = chronic obstructive pulmonary disease; cTnI = cardiac troponin I; ICU = intensive care unit.

## Competing interests

The authors declare that they have no competing interests.

## Authors' contributions

DAK performed literature research, contributed to protocol planning, was a primary data gatherer and drafted the manuscript. SC participated in data collection and statistical analysis. VN performed statistical analysis. LB participated in data collection. YA supervised research, planned protocol and edited the article.

## Figures and Tables

**Table 1:** Background characteristics of study population, according to elevated troponin result

	All (n = 128)^a^	cTnI positive (n = 35)^a^	cTnI negative (n = 93)^a^	p-value
Age (years)	53.9 ± 19	58.7 ± 18.6	52.1 ± 19	0.08
Older than 65 years	49 (38)	15 (43)	34 (37)	0.51
Male	68 (53.1)	19 (54.3)	49 (52.7)	0.87
APACHE II	15.3 ± 8.9	22.6 ± 10	12.7 ± 6.8	<0.001^b^
Cause of admission				
Sepsis	46 (35.9)	19 (54.3)	27 (29)	0.008
Respiratory failure	35 (27.3)	6 (17.1)	29 (31.2)	0.112
Poisoning/drug overdose	10 (7.8)	3 (8.6)	7 (7.5)	0.665
GI hemorrhage	6 (4.7)	2 (5.7)	4 (4.3)	0.844
Miscellaneous	31 (24.2)	5 (14.3)	26 (28)	0.108
Parameters upon admission				
Maximal temperature	37.9 ± 1.1	38.2 ± 1.3	37.8 ± 1	0.03^b^
Minimal mean arterial pressure	70.6 ± 21.7	63.2 ± 15.4	73.2 ± 23	0.02
White blood cell count (×1000)	14.9 ± 8.5	15.6 ± 7.8	14.5 ± 8.8	0.54
Creatinine (μmol/l)	142 ± 133	177 ± 106	124 ± 142	<0.001^b^
Use of pressors	48 (37.5)	17 (48.6)	31 (33.3)	0.11
28-day mortality	24 (18.8)	15 (42.9)	9 (9.7)	<0.001
Length of stay (ICU)	3.3 ± 28.5	6.4 ± 6.6	2.2 ± 33.1	0.07^b^
Days on mechanical ventilation	3.7 ± 5.4	3.2 ± 5.1	5.4 ± 6.0	0.004^b^

^a^Numbers are n, percentages (in parentheses) or mean ± SD, as appropriate. ^b^Man-Whitney test was applied due to abnormality of the data distribution. APACHE, Acute Physiology and Chronic Health Evaluation; ICU, intensive care unit. GI, Gastrointestinal hemorrhage

**Table 2:** Background characteristics of study population, according to outcome

	All (n = 128)^a^	Alive (n = 104)^a^	Dead (n = 24)^a^	p value
Age (years)	53.9 ± 19	51.6 ± 19.4	64 ± 13.6	0.001
Male	68 (53.1)	57 (54.8)	11 (45.8)	0.427
APACHE II	15.3 ± 8.9	13.6 ± 7.5	22.7 ± 10.9	<0.001^b^
Cause of admission				
Sepsis	46 (35.9)	34 (32.7)	12 (50)	0.111
Respiratory failure	35 (27.3)	27 (26)	8 (33.3)	0.465
Poisoning / drug overdose	10 (7.8)	10 (9.6)	0	0.206
GI hemorrhage	6 (4.7)	5 (4.8)	1 (4.2)	1.0
Miscellaneous	31 (24.2)	28 (26.9)	3 (12.5)	0.137
Parameters upon admission				
Maximal temperature	37.9 ± 1.1	37.8 ± 1.1	38.1 ± 1.4	0.111^b^
Minimal mean arterial pressure	70.6 ± 21.7	73.1 ± 22.1	59.5 ± 15.9	0.006
White blood cell count (×1000)	14.8 ± 8.5	14.7 ± 8.1	15.4 ± 10.2	0.723
Creatinine (μmol/l)	133 ± 133	133 ± 142	159 ± 88	0.004^b^
Use of pressors	48 (37.5)	34 (32.7)	14 (58.3)	0.02
cTnI > 0.7	35 (27.3)	10 (19.2)	15 (62.5)	<0.001
Length of stay (ICU)	3.3 ± 28.5	2.7 ± 31.5	6.3 ± 5.9	0.045^b^
Days on mechanical ventilation	3.7 ± 5.4	3.1 ± 5.2	6 ± 5.7	<0.001^b^

^a^Numbers are n, percentages (in parentheses) or mean ± SD, as appropriate. ^b^Man-Whitney test was applied due to abnormality of the data distribution. APACHE, Acute Physiology and Chronic Health Evaluation; ICU, intensive care unit. GI, Gastrointestinal hemorrhage

**Table 3:** Results of logistic regression analysis for mortality (n = 128)

Variable	Coefficient	Standard error	OR	95% CI of OR	p-value
cTnI	1.04	0.6	2.82	0.87–9.2	0.085
APACHE II	0.09	0.03	1.094	1.02–1.17	0.009
